# Biomarker Discovery in Subclinical Mycobacterial Infections of Cattle

**DOI:** 10.1371/journal.pone.0005478

**Published:** 2009-05-08

**Authors:** Meetu Seth, Elise A. Lamont, Harish K. Janagama, Andrea Widdel, Lucy Vulchanova, Judith R. Stabel, W. Ray Waters, Mitchell V. Palmer, Srinand Sreevatsan

**Affiliations:** 1 Department of Veterinary Population Medicine, University of Minnesota, St Paul, Minnesota, United States of America; 2 Department of Veterinary and Biomedical Sciences, University of Minnesota, St Paul, Minnesota, United States of America; 3 National Animal Disease Center, United States Department of Agriculture, Ames, Iowa, United States of America; University of Hyderabad, India

## Abstract

**Background:**

Bovine tuberculosis is a highly prevalent infectious disease of cattle worldwide; however, infection in the United States is limited to 0.01% of dairy herds. Thus detection of bovine TB is confounded by high background infection with *M. avium* subsp. *paratuberculosis*. The present study addresses variations in the circulating peptidome based on the pathogenesis of two biologically similar mycobacterial diseases of cattle.

**Methodology/Principal Findings:**

We hypothesized that serum proteomes of animals in response to either *M. bovis* or *M. paratuberculosis* infection will display several commonalities and differences. Sera prospectively collected from animals experimentally infected with either *M. bovis* or *M. paratuberculosis* were analyzed using high-resolution proteomics approaches. iTRAQ, a liquid chromatography and tandem mass spectrometry approach, was used to simultaneously identify and quantify peptides from multiple infections and contemporaneous uninfected control groups. Four comparisons were performed: 1) *M. bovis* infection versus uninfected controls, 2) *M. bovis* versus *M. paratuberculosis* infection, 3) early, and 4) advanced *M. paratuberculosis* infection versus uninfected controls. One hundred and ten differentially elevated proteins (*P*≤0.05) were identified. Vitamin D binding protein precursor (DBP), alpha-1 acid glycoprotein, alpha-1B glycoprotein, fetuin, and serine proteinase inhibitor were identified in both infections. Transthyretin, retinol binding proteins, and cathelicidin were identified exclusively in *M. paratuberculosis* infection, while the serum levels of alpha-1-microglobulin/bikunin precursor (AMBP) protein, alpha-1 acid glycoprotein, fetuin, and alpha-1B glycoprotein were elevated exclusively in *M. bovis* infected animals.

**Conclusions/Significance:**

The discovery of these biomarkers has significant impact on the elucidation of pathogenesis of two mycobacterial diseases at the cellular and the molecular level and can be applied in the development of mycobacterium-specific diagnostic tools for the monitoring progression of disease, response to therapy, and/or vaccine based interventions.

## Introduction

Bovine tuberculosis (bovine TB), a zoonotic infection in cattle caused by the intracellular bacterium *Mycobacterium bovis* (*M. bovis*), is a highly prevalent infectious disease of cattle worldwide [1]. Human tuberculosis caused by *M. bovis* is underestimated and the evidence that animal-human and inter-human transmission can occur underscores the importance of undertaking control programs based on robust understanding of the pathogenesis of the disease [2], [3], [4]. Bovine TB causes a conservative annual loss of three billion dollars in the United States (U.S.) of which $3.5–4 million is attributed to the current “test and slaughter” eradication program [5].

Cattle of all ages are susceptible to *M. bovis* infection; however, older animals have an increased risk of infection [6], [7], [8]. In most cases, *M. bovis* infection leads to subclinical disease (95%) with rapid clinical onset occurring in only 5% of exposed animals. Although *M. bovis* is wide spread, bovine tuberculosis is limited to 0.01% of dairy herds in the U.S. This is in stark contrast to another mycobacterial disease of cattle, Johne's Disease (JD), which afflicts 68% of U.S. dairy herds (2007, USDA, report). Consequently, *M. bovis* detection is confounded by background infection with *M. paratuberculosis (MAP)*, the causative agent of JD. Current USDA surveillance for bovine TB fails to address the aforementioned issue. USDA testing entails a laborious multistep procedure involving the caudal fold test (CFT) and the comparative cervical test (CCT) or γ-interferon detection. Despite advancements in diagnostic approaches several problems persist: CFT lacks specificity for *M. bovis* and is often and fails to detect all diseased cattle, while the γ-interferon assay is costly and requires blood samples to be processed within 24 hours of collection. Although serological tests using recently refined antigens have offered promising solutions, these also fall short since early detection relies on an active humoral immune response, which may not occur in a significant number of animals until the latter stages of *M. bovis* infection.

Failure to detect bovine TB poses a significant zoonotic threat and may result in a substantial loss of resources due to loss of trading partners, testing costs, culling, and quarantine of animals, as well as emotional stress to cattle owners. Thus, improved tests are urgently needed to increase detection, prevent disease exposure, and differentiate *M. bovis* infections from other mycobacterial species [9].

In order to develop sensitive and specific assays for bovine TB it is necessary to understand the pathogenesis of mycobacterial infections at the molecular level. Mycobacterial diseases are intractable infections in which the bacteria persist in granulomas for decades without disease progression [10]. Granuloma formation in mycobacterial infections share several commonalities at the molecular level, which may lead to significant changes in circulating peptidomes.

Proteomics offers a new approach of addressing early detection of *M. bovis* by identifying novel biomarkers. One proteomics method is isobaric tag for relative and absolute quantification (iTRAQ), a liquid chromatography and tandem mass spectrometry technique that allows for simultaneous identification and quantification of peptides between multiple sample groups. The purpose of this study was to implement iTRAQ in use with serum samples prospectively collected from cattle experimentally infected with either *M. bovis* or *M. paratuberculosis* for discovery of potential biomarkers of infection and progression of disease. Sera were used since respective protein profiles are expected to change concomitantly with the dynamics of granuloma formation in early and chronic infection. The biomarkers identified by iTRAQ were then validated on individual sera. This is the first study where the experimental design addresses the effect of progression of mycobacterial infections in animals on the circulating peptidome.

## Results

### Serum protein profiling: MALDI – TOF

Serum protein profiles (*N* = 30) from *M. bovis* infected (*n* = 5) and control (*n* = 5) animals representing three time points pre- and post-infection (PI) were generated using standard MALDI-TOF and analyzed by FlexAnalysis and ClinProTool™ software. The analysis did not result in the identification of statistically significant proteins unique to infected animals at any PI time point. However, the identification of similar protein profiles among infected and control sera confirmed that pooling of equal amounts of sera for iTRAQ would not lead to any significant variability.

### LC/MS/MS analysis: Multiplex analysis of *M. bovis* infected sera

**Figure 1:**
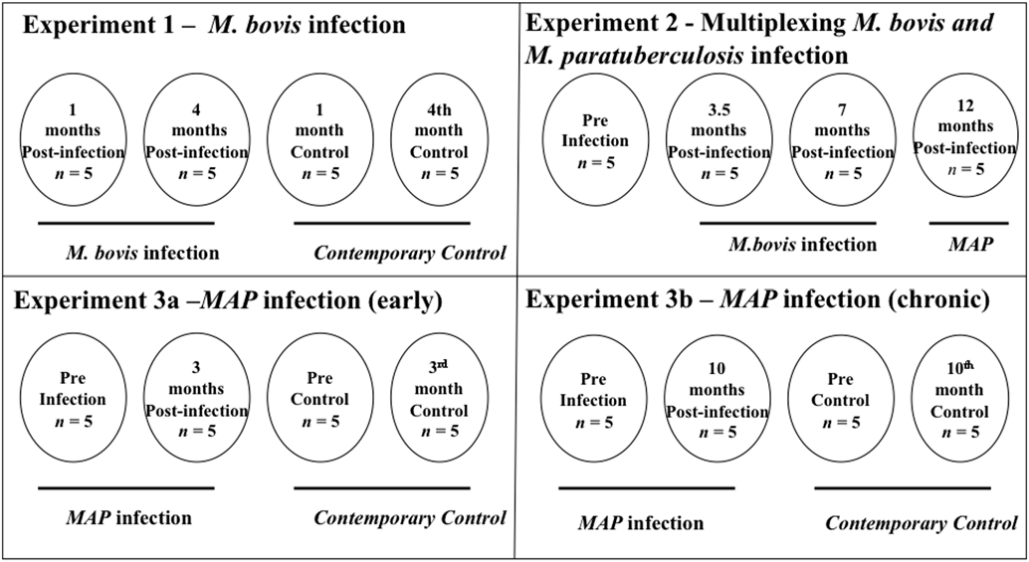
Experimental design used for four iTRAQ analyses with combinations of sera from *M. bovis* and/or *M. paratuberculosis* infections in calves. Shown in each quadrant is the experimental design for either *M. bovis* or *M. paratuberculosis* infections. Pre- and post-infection time points at which sera were collected and applied in iTRAQ analyses are identified. Numbers in brackets represent the numbers of sera from each group that was pooled for analysis. Experimental design also included contemporary controls.

**Figure 2:**
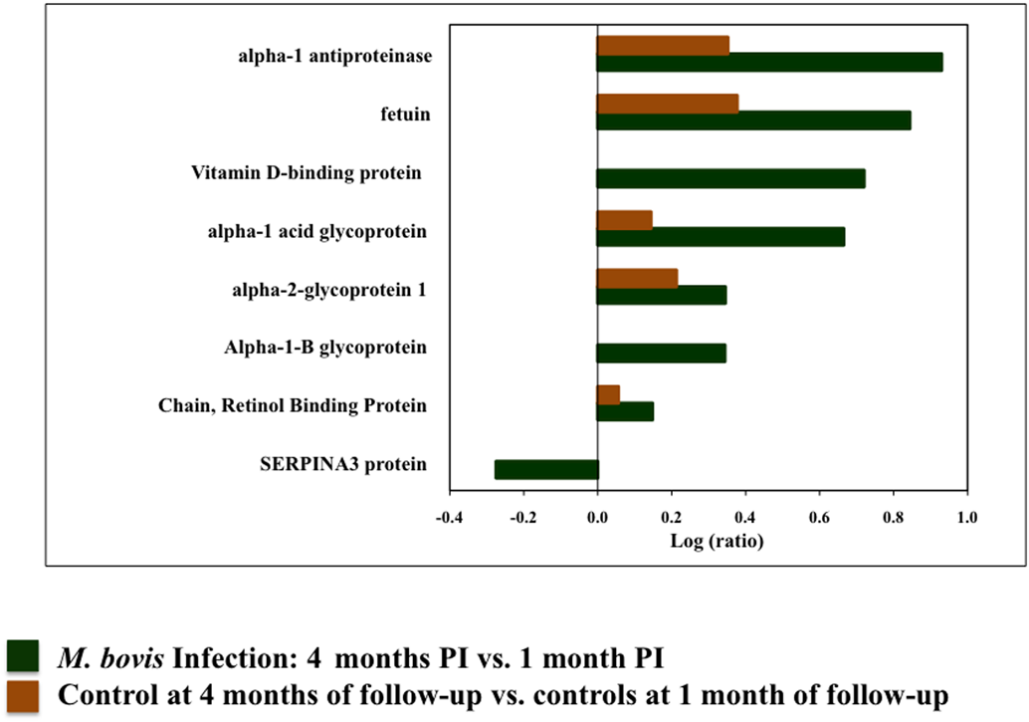
Biomarkers identified in *M. bovis* infection. The bar graph represents the log ratios of the proteins (*P*<0.05, EF<2) identified in *M. bovis* infected and control animals. The green bars represent log ratios of serum biomarkers between 4 months and 1 month PI with *M. bovis*. Shown in red are ratios in within contemporary controls over time. Levels of D-binding protein and alpha-1 B glycoprotein increased as *M. bovis* infection progressed, while that of serpina3 protein decreased. Serum amyloid A1 levels increased only in the control animals over time.

Sera from five animals at 1 and 4 months PI and uninfected controls were pooled and analyzed by iTRAQ (Figure 1). A total of 104 proteins were identified with 95% CI of which 81 carried 99% confidence. The log ratios of the reporter ions at 4 months PI versus 1 month PI was compared against uninfected controls. Twenty-eight proteins were differentially expressed in this comparison (*P*<0.05, EF<2) (Figure 2, Table 1).

**Table 1:** List of proteins identified in *M. bovis* infected animals and contemporary controls over a 5-month follow-up period.

Protein Id	Protein Name	1Ratio 115/114	3p-value	2Ratio 117/116	3p-value	4No. of peptides
95769204	alpha-1 antiproteinase, antitrypsin	8.50	0.00	2.24	0.00	15
344	fetuin	6.98	0.00	2.38	0.00	23
85701291	Vitamin D-binding protein precursor (DBP)	5.24	0.03	NI		2
124001426	alpha-1 acid glycoprotein	4.62	0.00	1.39	0.00	9
119892485	PREDICTED: similar to LOC539595 protein	2.69	0.00	1.80	0.00	1
86826324	Similar to leucine-rich alpha-2-glycoprotein 1	2.21	0.00	1.63	0.00	10
86823979	Alpha-1-B glycoprotein	2.20	0.00	NI		6
82697389	hypothetical protein LOC537301	2.14	0.00	NI		14
92097532	Similar to chromosome 6 open reading frame 82	1.62	0.02	NI		1
119915840	similar to coagulation factor XII A1 polypeptide	1.64	0.03	NI		3
146345493	Pulmonary surfactant-associated protein	1.60	0.03	1.43	0.00	1
59857769	inter-alpha (globulin) inhibitor H4	NI		0.78	0.01	3
75832056	apolipoprotein A-I	1.55	0.00	1.11	0.00	14
86438273	C-type lectin domain family 3, member B	1.52	0.01	1.91	0.02	1
93141264	Fibrinogen alpha chain precursor	NI		0.56	0.00	9
809399	Chain, Retinol Binding Protein	1.40	0.00	1.13	0.02	4
114019	Apolipoprotein C-II (Apo-CII) (ApoC-II)	1.37	0.00	NI		10
28189424	similar to beta 2-microglobulin	NI		0.82	0.01	6
81674721	Similar to Apolipoprotein D precursor	1.23	0.00	1.24	0.00	4
77735469	apolipoprotein M	1.21	0.00	NI		2
74267870	Transthyretin	NI		1.17	0.04	8
84201715	Similar to Apolipoprotein A-II precursor	NI		0.84	0.00	33
86438511	ApoN protein	1.17	0.00	1.15	0.00	7
135806	Prothrombin precursor (Coagulation factor II)	0.91	0.03	0.93	0.00	20
85057070	Apolipoprotein C-III	0.91	0.00	0.92	0.00	13
119914034	PREDICTED: similar to endopin 2B	0.81	0.00	1.08	0.04	11
74268120	Hemoglobin alpha chain	0.73	0.00	1.50	0.00	19
99028969	complement component 3	0.69	0.00	0.75	0.00	9
61816056	similar to keratinocyte differentiation-associated protein isoform 2	0.65	0.02	NI		3
114326282	transferrin	NI		1.63	0.01	2
81674551	Similar to serum amyloid A1	NI		3.08	0.00	2

1 115/114: ratio of the fold change of proteins in 4 months post *M. bovis* infection compared with one month post infection.

2 117/116: ratio of the fold change of protein biomarkers at 4 months post *M. bovis* infection compared with contemporary control at the same time point.

3 *p*-values are fold ratios of the different reporter ions with a significance of <0.05.

4 Number of peptides represent those identified with 99% confidence in the reporter ion quantification.

NI : not identified.

### Multiplex analysis of *M. bovis* and *M. paratuberculosis* infected sera

**Figure 3:**
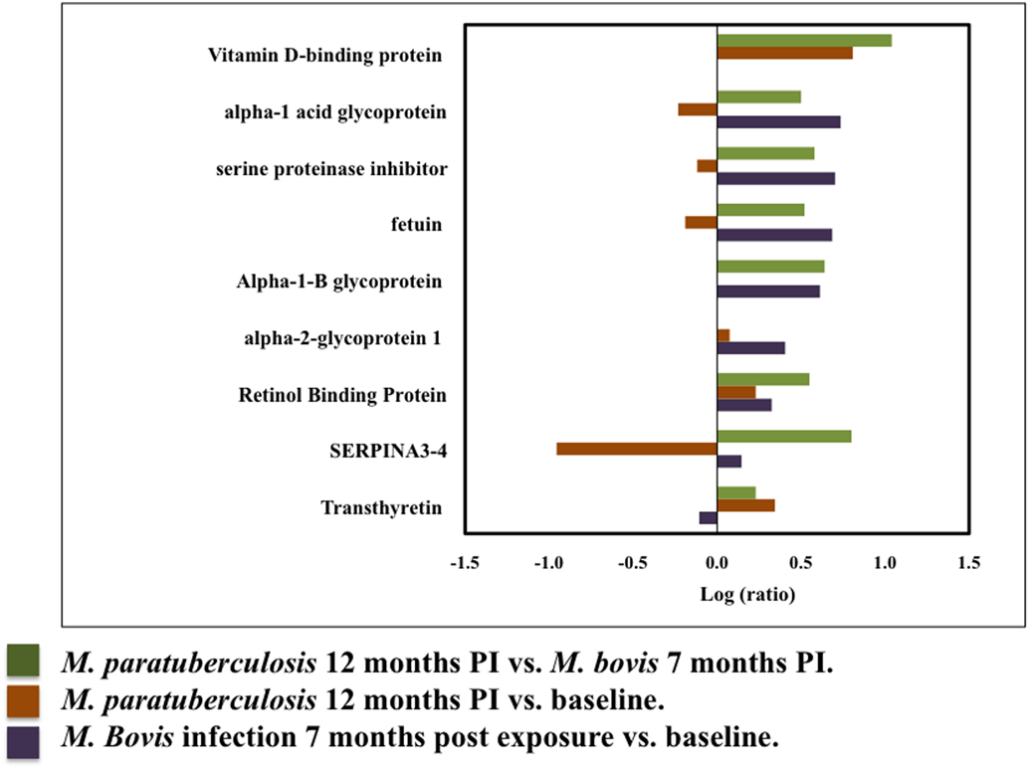
Biomarkers identified in *M. bovis* and *M. paratuberculosis* infection. The bar graph represents log ratios of serum proteins identified (*P-*value<0.05, EF<2) in *M. bovis* infected and *M. paratuberculosis* infected animals. The purple bars represent the log ratios of proteins following infection with *M. paratuberculosis* at 7 months PI vs. pre-infection. The brown bars represent log rations of proteins following infection with *M. bovis* at seventh month PI vs. pre-infection. The green bars represent log ratio of proteins at 12 months PI vs. *M. bovis* 7 months PI. Serum levels of D-binding protein and transthyretin were significantly increased in *M. paratuberculosis* infected animals compared with controls, while the serum levels of AMBP protein, alpha-1 acid glycoprotein, fetuin, and alpha-1B glycoprotein were elevated exclusively in *M. bovis* infected animals.

*M. bovis* infected sera (0, 3.5, and 7 months PI) and *M. paratuberculosis* sera (12 months PI) were pooled and analyzed. A total of 138 proteins were detected (CI = 95%) of which 97 carried a 99% confidence. The log ratios of the reporter ions at 7 months PI with *M. bovis* and 12 months PI with *M. paratuberculosis* were analyzed against pre- *M. bovis* infection. Twenty-three proteins were differentially expressed in this comparison (*P*<0.05, EF<2) (Figure 3, Table 2).

**Table 2:** List of serum proteins identified in *M. bovis* infected animals at 7 months post-infection and 10 months post-infection in *M. paratuberculosis* infection.

Protein Id	Protein Name	1Ratio 114/116	3p-value	2Ratio 117/114	3p-value	3Ratio 117/116	3p-value	4No. of peptides
74354219	AMBP protein	14.38	0.01	NI		15.46	0.00	4
85701291	Vitamin D-binding protein precursor (DBP)	NI		6.42	0.00	11.00	0.14	4
124001426	alpha-1 acid glycoprotein	5.43	0.00	0.58	0.00	3.16	0.00	8
95769204	alpha-1antiproteinase, antitrypsin	5.03	0.00	0.76	0.00	3.84	0.00	21
344	fetuin	4.84	0.00	0.64	0.00	3.30	0.00	23
86823979	Alpha-1-B glycoprotein	4.09	0.00	NI		4.37	0.00	6
135806	Prothrombin precursor (Coagulationfactor II)	3.71	0.01	NI		6.26	0.01	23
115495209	hypothetical protein LOC531420	2.88	0.02	NI		2.19	0.04	1
119915491	similar to Complement C4-A precursor	2.70	0.00	0.63	0.02	2.05	0.04	22
494449	Chain D, Platelet Factor 4	2.47	0.00	1.57	0.00	4.38	0.00	1
86826324	Similar to Leucine-rich alpha-2-glycoprotein	2.54	0.00	1.14	0.01	2.93	0.00	12
809399	Chain, Retinol Binding Protein	2.11	0.00	1.69	0.00	3.59	0.00	8
27806875	histidine-rich glycoprotein	1.54	0.02	0.42	0.00	0.67	0.01	1
75832056	apolipoprotein A-I	1.49	0.00	NI		1.34	0.00	14
81674721	Similar to apolipoprotein D precursor	NI		1.53	0.00	NI		4
121531628	SERPINA3–4	1.39	0.00	0.11	0.00	0.15	0.03	2
85057070	Apolipoprotein C-III	0.88	0.00	0.76	0.00	0.68	0.00	18
114019	Apolipoprotein C-II	0.84	0.01	0.59	0.00	0.50	0.00	9
119893035	Similar to alpha-2-macroglobulin	NI		0.79	0.02	0.76	0.02	7
151555847	Unknown (protein for MGC:157353)	0.79	0.03	NI		NI		1
74267870	Transthyretin	0.78	0.00	2.21	0.00	1.73	0.00	6
84201715	Similar to Apolipoprotein A-II precursor	0.76	0.00	0.90	0.00	0.70	0.00	30
498822	Alpha-2-antiplasmin	0.75	0.02	0.70	0.02	NI		1
61873128	similar to pancreas cationic pretrypsinogen isoform 1	0.72	0.00	NI		0.63	0.01	28
81674551	Similar to serum amyloid A1	0.69	0.00	0.37	0.00	0.26	0.00	3
119908847	similar to Fibrinogen beta chain precursor	0.65	0.02	NI		0.53	0.00	6
119915840	similar to coagulation factor XIII, A1 polypeptide	0.61	0.00	0.51	0.00	0.32	0.00	8
74268410	Alpha-1-antitrypsin	0.59	0.00	NI		NI		4
81	Apolioprotein E	NI		0.37	0.00	0.59	0.02	1

1 114/116: ratio of the fold change of proteins in *M. bovis* infected animals (7 months post-infection; 114) compared with pre-infection control animals (116).

2 117/114: ratio of the fold change of proteins in the *M. paratuberculosis* infection (10 months post-infection; 117) relative to *M. bovis* infection (7 months post-infection; 114).

3 117/116: ratio of the fold change of proteins in the *M. paratuberculosis* infection (10 months post-infection; 117) compared with pre-infection control animals (116).

3 *p*-value represents fold ratios of the different reporter ions with a significance of <0.05.

4 Number of peptides represent those identified with 99% confidence in the reporter ion quantification.

NI: Not Identified.

### Multiplex analysis of sera from early *M. paratuberculosis* infection

**Figure 4:**
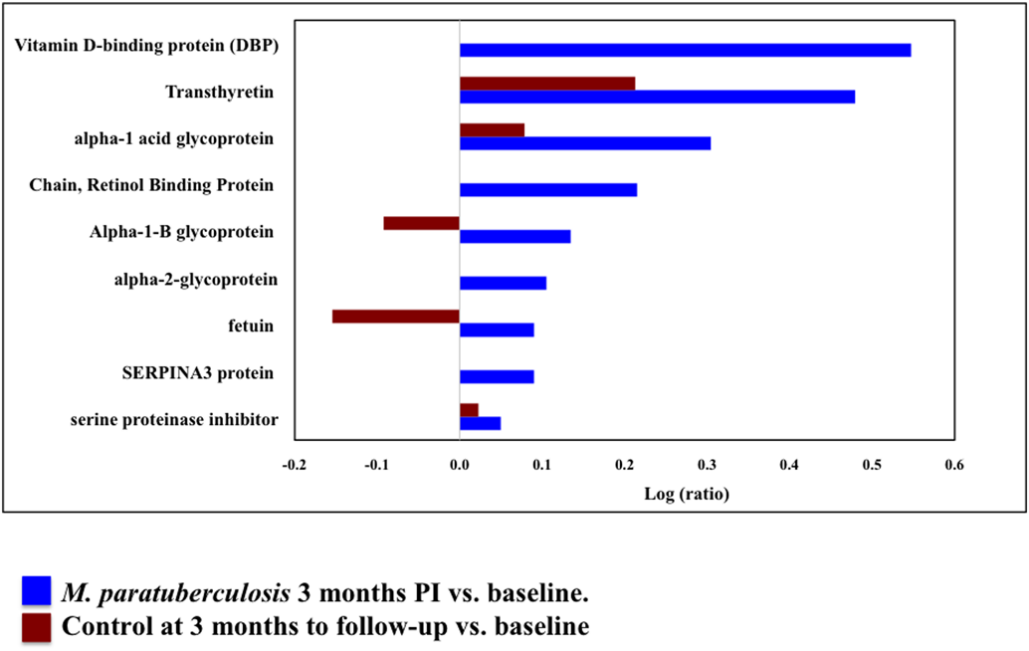
Biomarkers identified in early *M. paratuberculosis* infection. This bar graph represents log ratios of serum proteins (*P-*value<0.05, EF<2) identified in early (3-months to follow-up) *M. paratuberculosis* infected vs. control animals. The blue bars represent log ratios of proteins in animals infected with *M. paratuberculosis* over time (3 months PI vs. baseline). The red bars represent log ratios of the same proteins in control animals over time.

Sera from five *M. paratuberculosis* infected animals at baseline, 3 months PI, and uninfected controls were pooled and analyzed. A total of 146 proteins were identified (CI = 95%) of which 106 carried a 99% confidence. Twenty-eight proteins were differentially expressed in this comparison (*P*<0.05, EF<2) (Figure 4, Table 3).

**Table 3:** List of serum proteins identified in *M. paratuberculosis* infected calves (3 months post-infection) and contemporary controls compared with pre-infection control animals.

Protein Id	Protein Name	1Ratio 114/115	3p-value	2Ratio 116/117	3p-value	4No. of peptides
85701291	Vitamin D-binding protein precursor (DBP)	3.52	0.00	NI		15
74267870	Transthyretin	3.01	0.00	1.63	0.00	9
86438034	Similar to C1q and tumor necrosis factor related protein 5 (predicted)	2.32	0.01	1.99	0.01	2
75832056	apolipoprotein A-I	2.06	0.00	1.27	0.00	22
61680008	Chain B, Crystal Structure Of Bovine Plasma Copper-Containing Amine Oxidase	2.05	0.00	NI		7
94966811	alpha-1 acid glycoprotein	2.01	0.00	1.19	0.00	13
119915491	similar to Complement C4-A precursor	1.93	0.00	0.72	0.01	17
78045497	vitronectin	1.77	0.00	NI		1
27806743	alpha-1-microglobulin/bikunin	1.68	0.00	NI		4
809399	Chain, Retinol Binding Protein Complexed	1.63	0.00	NI		6
119892485	similar to LOC539595 protein	1.57	0.03	1.31	0.01	4
86823979	Alpha-1-B glycoprotein	1.36	0.00	0.80	0.00	12
86826324	Similar to leucine-rich alpha-2-glycoprotein 1	1.27	0.00	NI		14
99028969	complement component 3	1.27	0.00	1.15	0.00	6
86438511	ApoN protein	NI		1.22	0.00	6
95769204	alpha-1 antiproteinase, antitrypsin	NI		1.05	0.02	16
344	fetuin	1.23	0.00	0.70	0.00	11
86438018	SERPINA3 protein	1.23	0.04	NI		5
81674721	Similar to Apolipoprotein D precursor	1.15	0.02	1.31	0.00	4
95769204	alpha-1 antiproteinase, antitrypsin	1.12	0.00	1.05	0.02	16
84201715	Similar to Apolipoprotein A-II precursor	0.95	0.03	1.29	0.00	7
85057070	Apolipoprotein C-III	0.81	0.00	1.19	0.00	8
494	kininogen	0.69	0.00	NI		1
95147674	similar to Complement factor B precursor (C3/C5 convertase)	NI		0.81	0.01	2
494449	Chain D platelet factor 4	NI		0.72	0.02	1
135806	Prothrombin precursor (Coagulation factor II)	0.66	0.00	NI		14
1346006	Fibrinogen beta chain precursor	0.66	0.00	NI		1
74356489	HBG protein	0.50	0.00	NI		12
76641144	PREDICTED: similar to Apolipoprotein C-II	0.46	0.00	0.64	0.00	5
74268120	Hemoglobin alpha chain	0.36	0.00	0.34	0.00	11

1 114/115: ratio of the fold change of proteins in calves at 3 month post-infection with *M. paratuberculosis* (114) compared with their pre-infection sera (115).

2 116/117: ratio of the fold change of serum proteins in the control animals (3 months to follow-up; 116) compared with animals at pre-infection (117).

3 *p*-value represent fold ratio of the different reporter ions with a significance of <0.05.

4 Number of peptides represent those identified with 99% confidence in the reporter ion quantification.

NI: not identified.

### Multiplex analysis of advanced infection with *M. paratuberculosis*

**Figure 5:**
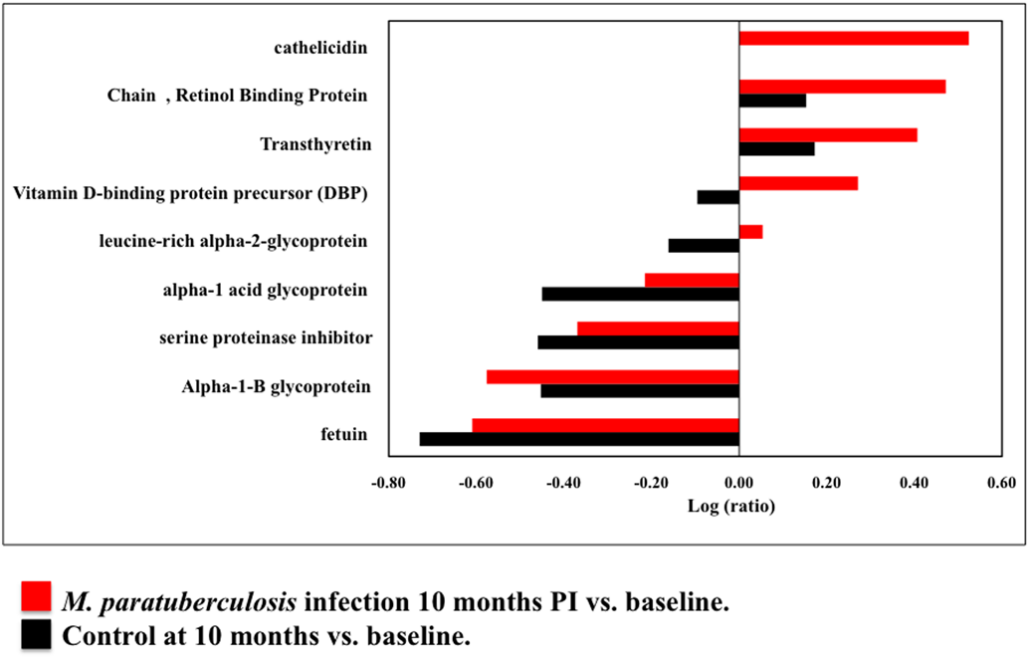
Biomarkers identified in advanced (10 months post infection) *M. paratuberculosis* infection. The bar graph represents log ratios of serum proteins (*P*-value<0.05, EF<2) identified in *M. paratuberculosis* infected (10 months PI) and control animals. Red bars represent log ratios of serum protein levels in *M. paratuberculosis* infected animals over time. The black bars represent the temporal trend in log ratios of the same proteins in control animals.

Sera from five *M. paratuberculosis* infected animals at baseline, 10 months PI and uninfected controls were pooled and analyzed. A total of 147 proteins were identified (CI = 95%) of which 109 carried a 99% confidence. Thirty-two proteins were differentially expressed in this comparison (*P*<0.05, EF<2) (Figure 5, Table 4).

**Table 4:** List of proteins identified in *M. paratuberculosis* infected animals at 10 months post-infection and contemporary controls relative to pre-infection.

Protein Id	Protein Name	1Ratio 117/115	3 *p*-value	2Ratio 116/114	3 *p*-value	4No of peptides
146345493	Pulmonary surfactant-associated protein B precursor	3.46	0.00	1.38	0.02	1
463	cathelicidin	3.34	0.04	NI		2
86438034	Similar to C1q and tumor necrosis factor related protein 5	3.03	0.01	NI		2
99028969	complement component 3	3.03	0.00	1.76	0.00	8
809399	Chain, Retinol Binding Protein	2.96	0.00	1.42	0.00	7
74267870	Transthyretin	2.55	0.00	1.48	0.00	11
119892485	similar to LOC539595 protein	2.10	0.00	0.83	0.00	4
76615300	similar to GCAP-I/guanylin	1.94	0.04	NI		2
84201715	Similar to Apolipoprotein A-II precursor	1.92	0.00	1.65	0.00	17
85701291	Vitamin D-binding protein precursor (DBP)	1.86	0.00	0.80	0.00	16
135806	Prothrombin precursor (Coagulation factor II)	1.52	0.00	1.36	0.04	23
75832056	apolipoprotein A-I	1.34	0.00	NI		22
51491835	ovarian and testicular apolipoprotein N	1.28	0.00	1.15	0.00	6
81674721	Similar to Apolipoprotein D	1.28	0.00	NI		5
85057070	Apolipoprotein C-III	1.27	0.00	1.54	0.00	17
27806743	alpha-1-microglobulin/bikunin	1.26	0.00	0.68	0.00	6
86826324	Similar to leucine-rich alpha-2-glycoprotein 1	1.13	0.00	0.68	0.00	15
114326282	transferrin	1.12	0.02	1.28	0.00	8
77735469	apolipoprotein M	0.86	0.00	0.76	0.00	5
86826424	Similar to Phosphatidylcholine-sterol acyltransferase	0.73	0.01	NI		1
151556981	Unknown (protein for MGC:157252)	0.73	0.00	0.64	0.00	1
77735387	fetuin B	0.72	0.00	0.55	0.00	2
27806703	CD44 antigen	NI		0.53	0.03	1
78045497	vitronectin	0.62	0.03	0.39	0.01	1
124001426	alpha-1 acid glycoprotein	0.60	0.00	0.35	0.00	16
119915494	similar to Complement C4-A	0.59	0.03	0.40	0.02	4
75812932	actin, cytoplasmic 2	0.50	0.00	NI		1
76641144	similar to Apolipoprotein C-II	0.43	0.00	0.76	0.00	8
95769204	alpha-1 antiproteinase, antitrypsin	0.42	0.00	0.34	0.00	27
86823979	Alpha-1-B glycoprotein	0.26	0.00	0.35	0.00	15
344	fetuin	0.24	0.00	0.18	0.00	40
81674551	Similar to serum amyloid A1	NI		0.20	0.00	3

1 117/115: ratio of the fold change of protein among animals at 10 months PI with *M. paratuberculosis* (117) against pre-infection (115).

2 116/114: ratio of the fold change of protein at 10 months to follow-up among contemporary controls(116) to pre-infection (114).

3 *p*-value represent fold ration of the different reporter ions with a significance of <0.05.

4 Number of peptides represent those identified with 99% confidence in the reporter ion quantification.

NI: not identified.

### Comparison of four iTRAQ experiments

Comparison of identified proteins in all four iTRAQs determined that both *M. bovis* and *M. paratuberculosis* infected sera showed elevated expression of Vitamin D Binding Protein (DBP) relative to age-matched contemporary controls. Vitamin DBP, serpina 3–4, transthyretin and retinol binding proteins were more abundant in *M. paratuberculosis* infection (Figure 3), while increased expression of alpha-1microglobulin, alpha-1 acid glycoprotein, serine proteinase inhibitor, fetuin and alpha-1 acid glycoprotein were detected in *M. bovis* infection with respect to *M. paratuberculosis* (Figure 3). Figure 3 shows *MAP* infection at 12 months compared to *M. bovis* infection at 7 months. As previously stated, vitamin DBP expression in *M. bovis* infected sera is comparable to baseline. Therefore, vitamin DBP is differentially expressed between *MAP* and *M. bovis* during late infection stages. Thus, we conclude that vitamin DBP expression is specific for *M. bovis* infection during mid-infection stages.

### Validation of protein biomarkers identified in infected animals

**Figure 6:**
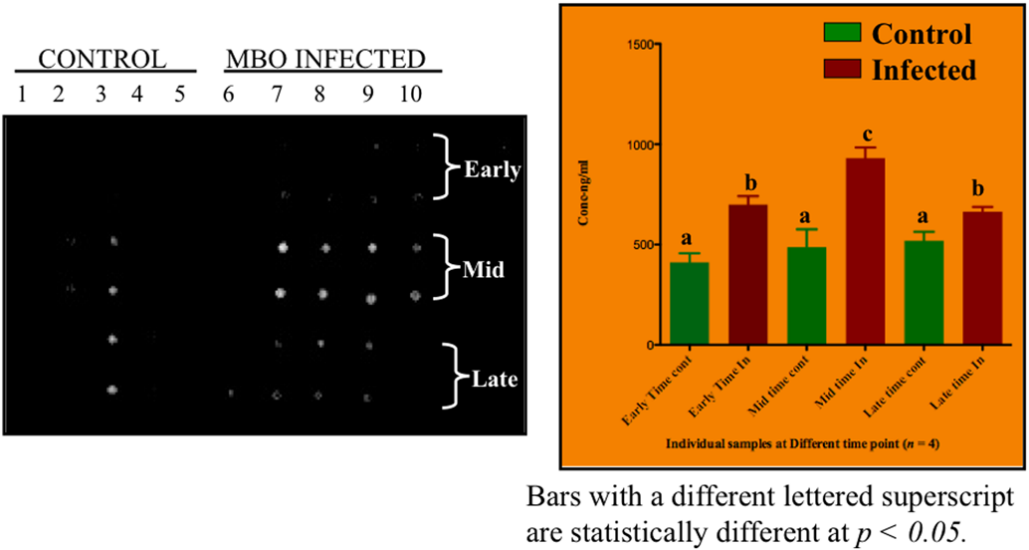
Validation of vitamin DBP in individual sera from *M. bovis* infected and control animals. Shown is a dot blot of levels of DBP in individual sera and the bar graph represents quantification of DBP in respective dots using densitometric analysis. Each spot contains 50-µg of total proteins from *M. bovis* infected and control sera. The first five lanes contain sera from control animals at first, third and fourth months. Lanes six to ten contains sera from *M. bovis* infection at 1, 3, and 4 months post infection. All sera were analyzed in duplicates.

In order to validate iTRAQ data, we performed western blot and dot blot analyses for vitamin DBP identified in *M. bovis* and *M. paratuberculosis* infection and alpha-1-microglobulin precursor, alpha-1-acid glycoprotein for *M. bovis* infection. The control group (*n* = 5) had similar expression in all animals at all time points of infection with the exception of animal 3. Interestingly, DBP was significantly elevated (*P*<0.05) during mid-infection (3 months PI) as compared to early (1 month PI) and late (4 months PI) infection or contemporary controls (Figure 6). Although animal 3 showed vitamin DBP reactivity, it did not display a similar expression profile as *M. bovis* infected animals. Therefore, animal 3 was considered an outlier. The dot-blot analysis for *M. paratuberculosis* infection (*n* = 4) showed vitamin DBP to be elevated at 9 months PI. Western blot analysis for alpha-1-microglobulin precursor and alpha-1-acid glycoprotein in *M. bovis* infection did not result in any significant differences between infected and control animals.

### Validation of DBP as a diagnostic biomarker for natural bovine tuberculosis infection

**Figure 7:**
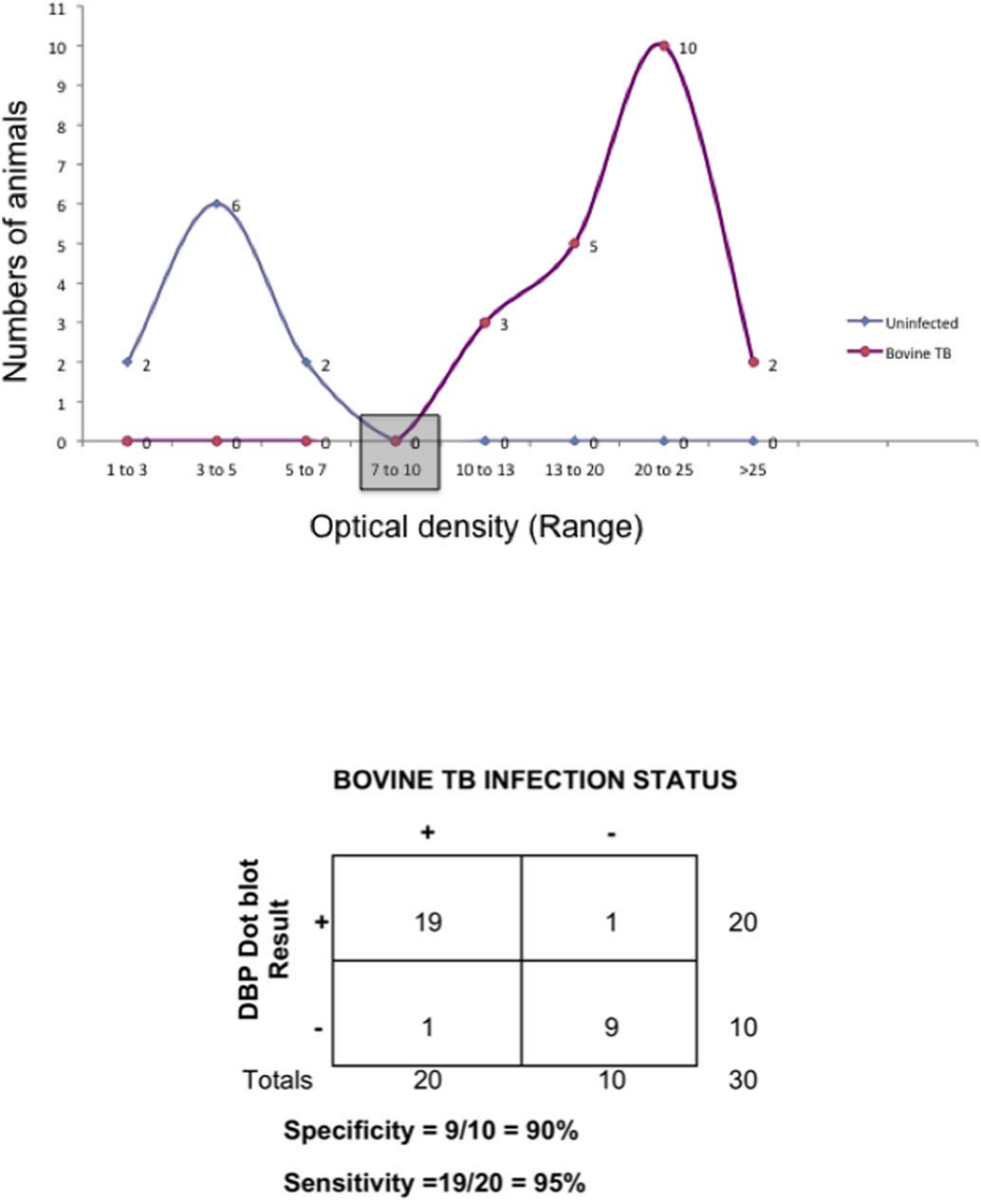
Validation of vitamin DBP in individual field sera from *M. bovis* infected and control animals. Frequency distribution of optical densities (OD) of vitamin DBP in sera of TB infected (*n* = 20) and control (*n* = 10) animals is shown. A clear bimodal distribution separates all infected animals from controls. Numbers of animals per OD range are shown at each node. A statistical “grey zone” using positive or negative OD average±2standard deviations led to misclassification of one animal in each category leading to 95% sensitivity and 90% specificity for DBP as diagnostic marker of bovine TB.

Inasmuch as iTRAQ of pooled sera identified that DBP could serve as a diagnostic biomarker, we performed a pilot validation on individual field sera collected from 20 bovine TB infected and 10 uninfected (control) animals using a dot blot approach. A frequency distribution analysis of optical densities identified a typical bimodal distribution with perfect separation of infected animals from uninfected controls (Figure 7). At 2 standard deviations from positive or negative means, an overlap and thus misclassification of <2 animals is expected to occur resulting in 95% sensitivity and 90% specificity. A larger validation study using sera collected from >650 field cases bovine TB and 150 uninfected animals is currently underway and is expected to provide more accurate measures of validity.

## Discussion

Despite the aggressive USDA test and slaughter protocol for bovine TB, it remains a significant economic and zoonotic threat. Bovine TB control and eradication depends on the development of tests capable of detecting early infection and discriminating bovine TB from *M. paratuberculosis*. In this report, we describe a proteomics approach, iTRAQ, to identify serum biomarkers for sub-clinical bovine TB and JD. These biomarkers can be applied in understanding pathogenesis and development of early diagnostic tools for both infections.

We identified several common and distinct serum biomarkers for bovine TB and JD. Our first finding suggests that LC/MS/MS approach on distinct biological replicates are reproducible [11]. We used two separate sets of *M. bovis* infected animals and were able to reproducibly show consistency in serum protein profiles. iTRAQ analysis requires ∼100 µg of protein for unambiguous consultation of serum peptidome after depleting for serum albumin, which represents >80% of the total proteins. Therefore iTRAQ analyses were performed on sera pooled from 5 animals at each infection stage. Our data demonstrated similar protein profiles among individual sera from infected and control animals using MALDI-TOF confirming that the pooling sera in equal amounts for iTRAQ did not lead to any significant variability between pooled samples. Next, we demonstrated that individual sera from infected animals were indeed positive for DBP indicating the validity of peptides identified by iTRAQ. Finally, the utility of DBP as diagnostic marker was demonstrated on individual sera from 20 bovine TB infected and 10 control animals with 95% sensitivity and 90% specificity.

We identified approximately 30 proteins (P<0.05, EF<2) in all iTRAQ comparisons. One biologically significant protein common to both bovine TB and JD was vitamin DBP. Vitamin DBP is approximately 49 kDa [12] and serves a number of functions in vivo, including vitamin D transportation and macrophage activation [13], [14], [15]. Since 1895, vitamin D has been shown to play a major role in mycobacterial diseases and was used to treat pulmonary tuberculosis in humans during the 1920 s [16]. The role of vitamin D as an anti-tuberculosis agent was recently demonstrated by Liu *et al.*, in which vitamin D expression via toll-like receptors 2/1 (TLR2/1) led to increased killing of intracellular *M. tuberculosis* by induction of cathelicidin, also known as LL-37 [17], [18]. Cathelicidins, a group of antimicrobial peptides that range from 12 to 80 amino acid residues, disrupt microbial membranes [19]. In our study cathelicidins were identified at greater levels in *M. paratuberculosis* infection at 10 months PI in comparison to controls, suggesting a possible role in antimycobacterial activity. These findings correlate with a recent *M. bovis* study conducted by Meade et al. in which several Toll associated molecules were identified in MBO infection.

Increase of DBP in cattle during early mycobacterial infections may occur due to the activation of the host's innate immune response. Therefore, we propose a common pathway involved in upregulation of vitamin DBP and cathelicidin in response to mycobacterial infections in cattle similar to that reported in humans [18].

In addition to DBP and LL-37, six acute phase proteins were identified in infected cattle. Acute phase proteins identified in this study include serine proteinase inhibitor (alpha-1-antitrypsin, AAT), fetuin, alpha-1 acid glycoprotein, complement C3 and alpha-1 beta glycoprotein. Since 1984, these acute-phase proteins have been investigated in relation to human TB. Grange *et al*, reported increased levels of eight acute-phase reactants (AAT, α2-macroglobulin, transferrin, α1-acid glycoprotein, C-reactive protein, ceruloplasmin, haptoglobin, and the third component of complement) in human serum from Indonesian patients with pulmonary tuberculosis [20]. Later in 1989, Wong *et al* measured these acute phase proteins in tuberculosis patients from Singapore [21]. Alpha1-acid glycoprotein is increased in response to systemic infection and has been shown to modulate apoptosis in bovine monocytes, probably through caspase-3 inhibition and caspase-9 activation [22]. AAT, the most abundant serine proteinase inhibitor, increases rapidly in response to inflammation or infection and has been shown to suppress *M. abscessus* infection of monocyte-derived macrophages [23]. Thus, we hypothesize that these acute phase proteins are integral to host defense in mycobacterial infections in cattle.

One other significant discovery related to *M. paratuberculosis* infection in cattle, in this study, was the identification of transthyretin (pre-albumin) and retinol binding protein. Transthyretin, a 55-kDa homotetramer in serum, is a transporter of thyroxine and tri-iodothyronine, as well as vitamin A (retinol or trans-retinoic acid) through association with retinol-binding protein [24], [25]. Retinoic acid has been demonstrated to stimulate and differentiate monocytes leading to the inhibition of *M. tuberculosis* multiplication in human macrophages [26]. Furthermore, retinoic acid is required for Ig class switching to IgA, which is ubiquitous in the mucosal immune system. Transthyretin is also considered a negative acute phase protein and is decreased in inflammation, malignancy, cirrhosis of the liver and protein wasting diseases due to reduced synthesis. Transthyretin is used as a specific clinical indicator for nutritional risk management of diseases such as HIV/AIDS, renal disease, diabetes, pneumonia and cancer. It has also been identified as a negative biomarker for tuberculosis patients in humans [27]. Since malnutrition is the most common symptom observed in MAP infected animals, we believe that a transthyretin based screening test can be used to identify and monitor progression of JD infection in animals. In addition, increased levels of retinol binding protein supports our hypothesis of a vitamin A based host response to *M. paratuberculosis* infection. Vitamin A may act as a stimulating factor for monocyte differentiation, thus inhibiting *M. paratuberculosis* multiplication in macrophages similar to that demonstrated in *M. tuberculosis* [26].

Biomarker discovery by LC/MS/MS based method was reproducible as previously documented [11]. This method is also highly sensitive and proteins can be identified in femtomole quantities and reaching sensitivity up to zeptomole (10^−21^ M) amounts [28], [29]. Thus validation of some of the identified peptides will require sensitive detection tools. ELISA may offer optimal sensitivity; however, lack of availability of bovine specific antibodies for different proteins is one major hurdle for ELISA development. Large-scale validation studies utilizing ELISA for several differentially expressed proteins in bovine TB and JD infected and control field sera are currently underway and should provide robust specificity data for the utility of individual and combinations of biomarkers in the diagnosis of mycobacterial disease among animals. It is likely that our approach can be extended to discover biomarkers for diagnosis or therapeutic monitoring of human mycobacterial infections.

In conclusion, we identified multiple biomarkers for bovine TB and JD using high-resolution proteomics approaches. Further validations using field samples are underway. Five different proteins identified common to both mycobacterial infections can help us in understanding the pathogenesis of these mycobacterial infections at the cellular and molecular levels. Elucidation of *M. bovis* disease progression as it relates to identified biomarkers in early infection could aid in tracking therapy/vaccine interventions as well as eradication programs in the U.S. and worldwide.

## Materials and Methods

### Sample source

Sera were obtained from calves infected with *M. bovis 1315* [30] or *M. paratuberculosis* K-10 [31] at the National Animal Disease Center (Ames, IA). All cattle used in this study were housed according to institutional guidelines and approved animal care and use protocols at the National Animal Disease Center, Ames, Iowa (NADC). For *M. bovis* challenge studies, cattle were housed in a biosafety level 3 (BL-3) facility. *M. bovis* infected sera were collected at baseline and prospectively every month post infection (PI) until 5 months, while *M. paratuberculosis* sera were collected prior to infection and every month for 12 months PI [32]. Experimental models included all disease associated immune response parameters, clinical signs, and lesion characteristics identified at necropsy [33]. Field samples of sera from bovine TB or MAP infected animals were obtained from serum repositories at National Veterinary Services Laboratory or from routine sample acquisitions into Minnesota Veterinary Diagnostic Laboratory, respectively. Bovine TB infection status of animals was confirmed by a combination of antemortem caudal fold test and histology of lesions at necropsy.

### Serum protein profiling using MALDI-TOF

Sera from infected (n = 3) and control animals (n = 3) representing different time points (1, 3, and 4 months PI) were Zip-Tip processed, desalted and spotted on the MALDI-Target [34], per manufacturer's instructions (Millipore, Billerica, MA). These samples were also profiled after organic depletion of abundant proteins using high performance liquid chromatography acetonitrile (ACN) (Sigma, St. Louis, MO).

### MALDI-TOF analysis

After the sample was dried on the MALDI target, profiling was done with the Bruker Biflex III (Bruker Daltonics, Billerica, MA). MALDI-TOF mass spectrometer (MS) was operated in linear mode at a laser power setting of 38±1% attenuation [34]. Five hundred laser shots were collected per sample while changing the spot at least ten times during data acquisition. The instrument was externally calibrated with the +1 and +2 charge state of cytochrome C (Sigma, St. Louis, MO). The spectra were internally calibrated with consistent protein peaks of *m/z* values of 8330 and 4165. Peaks between 2 kD–20 kD mass ranges were analyzed by Bruker XTOF 5.1.1 processing software (Sigma, St. Louis, MO). The raw data were smoothed (Golay-Savitzky formula using 15 points) and backgrounds were subtracted. The peaks were labeled and peak intensity lists were generated and compared.

### Sample preparation for LC/MS/MS

Sera from five animals **each** infected with *M. bovis* and *M. paratuberculosis* and contemporaneous controls were pooled in equal concentrations (100 µl each at 100 µg/µl) for four separate iTRAQ experiments - 1) *M. bovis* infection vs. contemporary controls, 2) *M. bovis* vs. *M. paratuberculosis* infection, 3) early and 4) chronic *M. paratuberculosis* infection vs. uninfected control (Figure 1). Due to required protein concentrations (80–100 µg/µL) for iTRAQ analysis, sera samples were pooled to obtain sufficient yield. Protein quantities were estimated using the bicinchoninic acid (BCA) protein assay (Pierce, Rockford, IL). A modified ACN method was used to deplete abundant proteins and concentrate limited proteins (31). Briefly, 100 ml of 1∶5 saline diluted sera were mixed with 200 µl of high performance liquid chromatography (HPLC) grade ACN (Sigma, St. Louis, MO) to a final concentration of 67%. Samples were vortexed and allowed to stand at room temperature for 30 min., then centrifuged at 13×1000 *g* for 10 min. in Eppendorf Minifuge tubes (Eppendorf, Westbury, NY). Supernatants were collected and lyophilized in a speed vacuum centrifuge. The lyophilized samples were dissolved in 20 µL of saline and protein concentration was estimated using BCA protein assay (Pierce, Rockford, IL). Sera were dialyzed against 20 mM triethyl ammonium bicarbonate buffer (TEAB) in a Slide-A-Lyzer Dialysis Cassette 2000 (MWCO) (Pierce, Rockford, IL) and stored at −80°C until used. Albumin and IgG depletion were confirmed by resolving and visualizing proteins on a 4–20% gradient polyacrylamide gel (Pierce, Rockford, IL), as well as Western blot analysis using anti-bovine albumin (Bethyl, Montgomery, TX) and anti-bovine IgG (H+L) antibodies (KPL, Gaithersburg, MD).

### Isobaric labeling

Approximately 80 µg of ACN depleted serum samples were labeled with iTRAQ reagents 114, 115, 116 and 117 following manufacturer's recommendations (Applied Biosystem (ABI), Foster City, CA). Briefly, the samples were freeze-dried and proteins were resuspended in dissolution buffer (0.5 M TEAB pH 8.5) and 0.1% SDS. Resuspended proteins were reduced, alkylated and digested with trypsin at 37°C overnight. Peptides were labeled with iTRAQ reagents at lysine and arginine amino terminal groups. The labeled peptides were pooled, dried and resuspended in 0.2% formic acid. The resuspended peptides were passed through Oasis® MCX 3CC (60 mg) extraction cartridges per manufacturer recommendations (Waters Corporation, Milford, MA) for desalting prior to strong cation exchange (SCX) fractionation.

### SCX Fractionation

The eluted peptides were dried and dissolved in 350 µl of SCX buffer A (20% v/v ACN and 5 mM KH_2_PO_4_ pH 3.2, with phosphoric acid) and fractionated using a polysulfoethyl A column (150 mm length×1.0 mm ID, 5 µm particles, 300 Å pore size) (PolyLC Inc., Columbia, MD) on a magic 2002 HPLC system (Michrom BioResources, Inc., Auburn, CA). Peptides were eluted by running a 0–20% buffer B gradient for greater than 55 min. and 20%–100% buffer B (20% v/v ACN, 5 mM KH_2_PO_4_ pH 3.2, 500 mM KCL) for 20 min. at a column flow rate of 50 µl/min. Thirty fractions were collected at 3 min. intervals, and each fraction was lyophilized. Fractions that showed mAU_280_>2 were analyzed by LC-MS/MS.

### Mass Spectrometry LC-ESI/MS/MS

Fractions were reconstituted in reversed-phase load buffer (10 mM phosphate buffer) and analyzed by LC-ESI/MS/MS [34] on a QSTAR Pulsar I-Quadrupole TOF MS using Analyst QS software. Briefly, peptides were loaded onto a LC Packing C18 cartridge and desalted with loading buffer (98∶2, water∶ acetonitrile, 0.1% formic acid) for 17 min. at 35 µl/min. Peptides were eluted by running an increasing ACN concentration gradient (5 to 35%) for greater than 47 min. Tandem mass spectral data were acquired in 3 s intervals using the information dependent acquisition (IDA) mode.

### Data Analysis and Interpretation

Protein pilot Software™ 2.0 and 2.0.1 (Applied Biosystems Inc., Foster city, CA) and the nr_bos_CTM_20070802 FASTA database were used to identify labeled peptides and determine relative abundance at a >95% confidence interval (CI). A minimum identification of at least two unique peptides per protein was used as a cutoff for protein analysis (P<0.05 and an error factor (EF) of <2). The EF expresses the 95% CI for an average ratio (EF = 10 ^95%confidence interval^, where 95% CI = (ratio×EF)−(ratio/EF)). Relative abundance of identified proteins was log_10_ transformed for further analysis.

### Immunoblotting

Approximately 50 µg of crude serum was dissolved in SDS-PAGE loading buffer, heated to 99°C in a thermal cycler (Eppendorf, Westbury, NY), and resolved on a 4–20% polyacrylamide-SDS gradient gel (Pierce, Rockford, IL). Resolved proteins were electrophoretically transferred onto a nitrocellulose membrane followed by overnight blocking in 0.05% TBE containing 5% skim milk. Primary antibodies used to detect vitamin DBP (R&D Systems, Minneapolis, MN) and alpha-1-microglobulin precursor (Gene Tex, San Antonio, TX) were mouse anti-human monoclonal antibodies. Anti mouse anti rabbit detection antibodies were conjugated to horseradish peroxidase (HRP). Primary antibody used to detect alpha-1-acid glycoprotein was rabbit anti-bovine conjugated to HRP (ICL, Newberg, OR).

In dot blot analyses, 0.5 µl of each serum sample was spotted in duplicate and dried on a nitrocellulose membrane. The nitrocellulose membrane was blocked using 0.05% TBE blocking buffer containing 0.5% skim milk for 1 hr., and washed with 0.05% PBST five times in 5 min. increments. Aforementioned primary and appropriate secondary antibodies were used in a similar fashion as western blotting. All immunoblots were developed using the chemiluminiscent detection kit (Pierce, Rockford, IL).
